# Exploring Relationships between Boltzmann Entropy of Images and Building Classification Accuracy in Land Cover Mapping

**DOI:** 10.3390/e25081182

**Published:** 2023-08-09

**Authors:** Zhipeng Li, Tian Lan, Zhilin Li, Peichao Gao

**Affiliations:** 1 Faculty of Geosciences and Environmental Engineering, Southwest Jiaotong University, Chengdu 611756, China; zhipengli@my.swjtu.edu.cn (Z.L.); dean.ge@swjtu.edu.cn (Z.L.); 2 Faculty of Geographical Science, Beijing Normal University, Beijing 100875, China; gaopc@bnu.edu.cn

**Keywords:** image quality, image complexity, building classification, classification accuracy, Boltzmann entropy, land cover mapping

## Abstract

Remote sensing images are important data sources for land cover mapping. As one of the most important artificial features in remote sensing images, buildings play a critical role in many applications, such as population estimation and urban planning. Classifying buildings quickly and accurately ensures the reliability of the above applications. It is known that the classification accuracy of buildings (usually indicated by a comprehensive index called F1) is greatly affected by image quality. However, how image quality affects building classification accuracy is still unclear. In this study, Boltzmann entropy (an index considering both compositional and configurational information, simply called BE) is employed to describe image quality, and the potential relationships between BE and F1 are explored based on images from two open-source building datasets (i.e., the WHU and Inria datasets) in three cities (i.e., Christchurch, Chicago and Austin). Experimental results show that (1) F1 fluctuates greatly in images where building proportions are small (especially in images with building proportions smaller than 1%) and (2) BE has a negative relationship with F1 (i.e., when BE becomes larger, F1 tends to become smaller). The negative relationships are confirmed using Spearman correlation coefficients (SCCs) and various confidence intervals via bootstrapping (i.e., a nonparametric statistical method). Such discoveries are helpful in deepening our understanding of how image quality affects building classification accuracy.

## 1. Introduction

Land cover datasets are important for studies on various subjects, including climate change [1], biodiversity conservation [2], ecosystem assessment [3] and urbanization assessment [4]. In recent decades, a variety of land cover datasets have been developed from remote sensing images with resolutions ranging from meters to kilometers, such as FROM-GLC10 [5], GLC 30 [6], MODIS [7] and GLC-2000 [8]. As one of the most common artificial features in land cover datasets, buildings play a critical role in population estimation [9], urban planning [10] and many other applications [11,12,13]. High-quality building data ensure the reliability of such applications. Usually, land cover building data are acquired from remote sensing images using automatic classification methods, and the classification accuracy of buildings is greatly affected by image quality. Therefore, some researchers have conducted a variety of studies on how image quality affects the applications of images [14,15,16,17]. More precisely, Roberts et al. [14] point out that remote sensing image quality plays an important role in assessing the image fusion process and can help in exploring which fusion method incorporates more texture information while retaining spectral information. Xia et al. [15] noticed that image quality is the key to successful image application, which provides the basis for classification, segmentation, etc. Li et al. [16] found that image quality assessment is widely used in applications such as image denoising, image deblurring and image fusion, and they conducted factor analysis and cluster analysis to assess the robustness of 21 commonly used non-reference image quality evaluation metrics in terms of accuracy, monotonicity and consistency. Bishop et al. [17] noted that image quality can be used to assess detection and classification performance. However, no explicit or quantitative relationships have been found between image quality and building classification accuracy.

There are a variety of no-reference metrics describing image quality. Among them, some metrics (e.g., root of mean square error metric (RMSE) [18], average gradient (AG) [19], signal-to-noise ratio (SNR) [20], Shannon entropy [21]) consider compositional information, while others (e.g., metrics based on the gray-level co-occurrence matrix [22], metrics based on the Sobel gradient [23], Boltzmann entropy [24]) consider configurational information. It should be explained here that the compositional information of a remote sensing image captures the composition of the image (i.e., the permutation of pixels), while configurational information captures the distribution of pixels in the image (i.e., the combination of pixels). Recently, some studies have been conducted to explore relationships between image properties and classification accuracy [25,26,27,28], such as those between land pattern and accuracy. It is reported that configurational information is very important to image quality [29]. Recently, some researchers have evaluated the existing metrics for describing configurational information, and a mathematical model between Boltzmann entropy (simply called BE in this study) and lossless compression ratio has been constructed [30]. In fact, Boltzmann entropy has full thermodynamic consistency [31], and it has been regarded as the most powerful metric for describing the configurational information of remote sensing images [32].

Regarding the methods for the building classification of remote sensing images, many traditional methods are available, such as K-Nearest Neighbors (KNNs) [33], support vector machines (SVMs) [34], random forest (RF) [35], etc. However, with the improvement in spatial resolution in remote sensing images, these traditional metrics are gradually being replaced by deep-learning-based methods, such as U-Net [36], SegNet [37] and DeepLabV3+ [38]. In this study, Boltzmann entropy is selected as the most appropriate metric for image quality, while DeepLabV3+ is employed as a suitable method for building classification. In addition, F1 (i.e., a popular and widely used metric for evaluating building classification) is employed as the accuracy index. We aim to explore the potential relationships between the Boltzmann entropy of land cover remote sensing images and F1. The remainder of this article is as follows. Section 2 introduces the experimental data and metrics/methods for exploring the relationships. Section 3 presents and analyzes the experimental results. Some discussions and conclusions are given in Section 4.

## 2. Experimental Data, Methods and Strategy

### 2.1. Building Image Datasets

**Figure 1:**
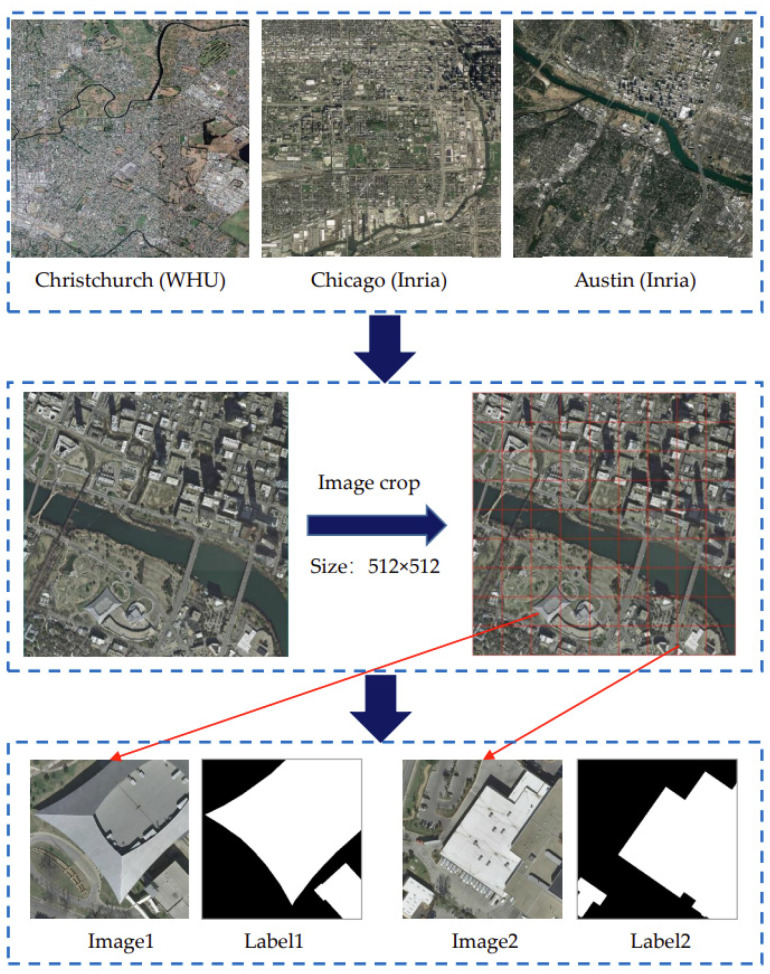
Overview of building images from WHU and Inria datasets.

Benefiting from the rapid development of remote sensing techniques and methods, some aerial very-high-resolution (VHR) building image datasets have been developed. Among them, four open-source datasets can be easily accessed, i.e., the Massachusetts [39], ISPRS [40], Inria [41] and WHU [42] datasets. The Massachusetts dataset includes 151 images with a size of 1500*1500 pixels and a spatial resolution of 1 m. It has been reported that the quality of this dataset is relatively poor (e.g., the average noise level of images is relatively high) [43]. The ISPRS dataset is small and only covers a 13 km^2^ area, which leads to few samples of buildings. The Inria and WHU datasets are newly developed ones with a spatial resolution of around 0.3 m covering more than 400 km^2^. To better explore the potential relationships between the Boltzmann entropy of images and building classification accuracy, the Massachusetts and ISPRS datasets are not employed. In this study, building images of three cities (i.e., Christchurch, Chicago and Austin) from the WHU and Inria datasets are employed for the experiments. An overview of the images of these four cities is shown in Figure 1. All the large images are clipped into small images with a size of 512*512 pixels for better training and testing results.

### 2.2. Boltzmann Entropy as a Metric for Image Quality

Entropy is a notion originating from thermodynamics to describe the lack of order that exists in a thermodynamic system [44,45], and Boltzmann proposed an explanation of entropy based on statistical physics. More precisely, a quantitative relationship model between entropy and the possible number of microstates in a system has been constructed [46]:

(1) $$S=k_{B}\log\left(W\right)$$

where S represents the Boltzmann entropy of a system; W is the possible number of microstates; $k_{B}$ is the Boltzmann constant (1.38*10^−23^ J/K).

Although the theoretical basis for using Boltzmann entropy to describe the configurational information of remote sensing images has been proven to exist, no feasible method for calculating entropy was proposed for a long period of time. This is because macrostates and their corresponding microstates are hard to define [47,48]. Recently, some researchers have proposed a feasible method through a landscape mosaic represented by gradients [29]. The key objective of such a method is to determine the macrostates of images and calculate the number of microstates.

Before illustrating the details of macrostates and microstates, the size of the original images should be explained. In this study, C and L are two variables representing the height and length of images (i.e., the number of pixels in the row and column of the image).

**Figure 2:**
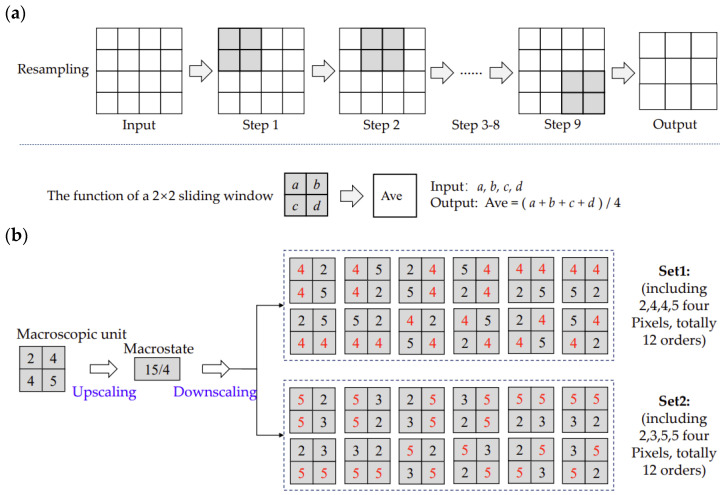
Macrostates, microstates and macroscopic units of an example image (modified from [29]). (**a**) The resampling process for acquiring macroscopic units; (**b**) the microstate and all possible microstates for a macroscopic unit.

In terms of macrostates, Gao et al. [29] gave two detailed definitions: (1) images with reduced resolution; and (2) images with a resolution of (C−1)*(L−1). Generally, the macrostates of images can be described by three parameters: maximum values, minimum values and averages. In practice, the size of a remote sensing image (i.e., C*L) is large. One solution is to separate a large image into small macroscopic units. For example, in Figure 2a, an original image is resampled by a moving a 2*2 window, and in this way, a variety of macroscopic units can be generated.

Under a macroscopic unit, a macrostate can be generated via upscaling, and then various microstates can be acquired via downscaling (see Figure 2b). In the example shown in Figure 2b, two multisets of microstates with the same maximum value, minimum value and average (i.e., 5, 2 and 15/4) can be generated via downscaling. Of all these microstates, only one has the same macroscopic unit. The details of determining multisets and the corresponding permutations for each multiset can be found in the paper by Gao et al. [29].

The total number of microstates for an original image (W) is the product of the numbers of microstates ($W_{u}$) for all individual macroscopic units:

(2) $$W_{\mathrm{image}}=\overset{n}{\underset{j=1}{\prod }}W_{u,j}$$

where $W_{u,j}$ is the $W_{u}$ calculated for the *j*th macroscopic unit. The formula for the calculation of $W_{u}$ is as follows:

(3) $$W_{u}=\overset{k}{\underset{i=1}{\sum }}M_{i}$$

where k is the number of multisets for a given microstate; $M_{i}$ is the number of permutations for each multiset. Therefore, the calculation formula of Boltzmann entropy for remote sensing images is as follows:

(4) $$S=k_{B}\log_{10}\left(W_{\mathrm{image}}\right)$$

where S is the relative Boltzmann entropy. In this study, the values of Boltzmann entropy refer to relative values, also called configurational entropy by Cushman [49].

### 2.3. DeepLabV3+ and F1 for the Classification of Buildings

**Figure 3:**
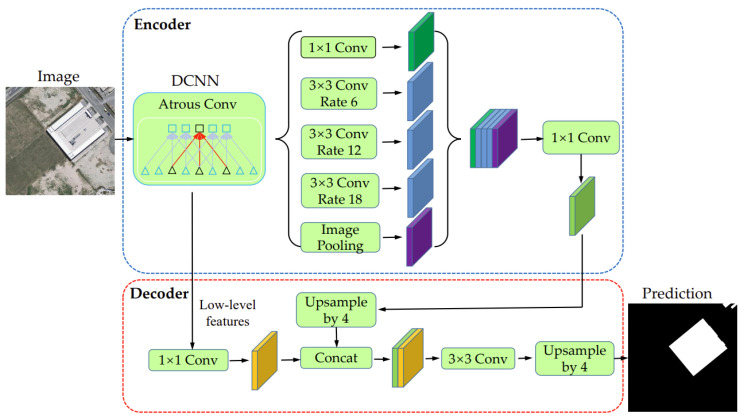
Schematic diagram of DeepLabV3+ (modified from [38]).

DeepLabV3+ is a semantic segmentation model based on a convolutional neural network proposed by Google Brain [38]. This model improves segmentation accuracy and calculation efficiency by introducing new modules and techniques (e.g., encoder–decoder structure and conditional randomness) when retaining the dilated convolution and multi-scale feature fusion of the original DeepLab series models (see Figure 3).

In terms of specific implementation, DeepLabV3+ uses deep neural networks such as ResNet as its encoding part, and uses the ASPP (Atrous Spatial Pyramid Pooling) module to further improve the receptive field. In the decoder part, DeepLabV3+ uses a series of deconvolution layers and bilinear interpolation layers combined with skip connection to fuse multi-scale features. In addition, DeepLabV3+ also uses conditional random fields to further optimize the segmentation results, resulting in more accurate pixel-level segmentation results. The main contribution of DeepLab V3+ is an efficient and accurate semantic segmentation model. The design of its hollow convolution and encoder–decoder structure enables the model to understand the semantic information of the image, and the innovations made in the deconvolution and bilinear interpolation allow the model to better incorporate multi-scale features.

F1 is a comprehensive index commonly used in classification models which comprehensively considers the “precision” and “recall rate” of the model. In the binary classification problem, “precision” (p) refers to the proportion of the number of positive samples correctly classified to the number of all positive samples predicted by the model, while “recall rate” (r) refers to the proportion of the number of positive samples correctly classified to the total number of actual positive samples. The formula for calculating F1 is as follows [50]:

(5) $$F1=2\times \frac{p\times r}{p+r}$$

### 2.4. A Strategy for Exploring the Potential Relationships between Boltzmann Entropy and F1 of Images

**Figure 4:**
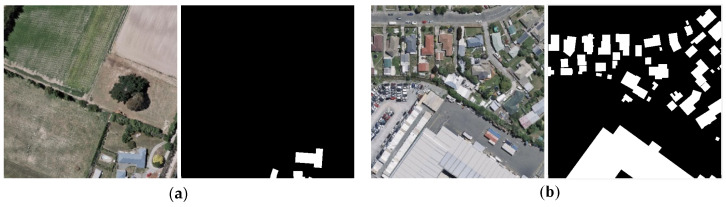
Two example images with different proportions of buildings. (**a**) The proportion of buildings is 2%; (**b**) the proportion of buildings is 27%.

F1 for building classification is mainly affected by the differences within the buildings themselves (intra-class differences) and the differences between buildings and non-buildings (inter-class differences). Intra-class differences are mainly due to the diverse spectrum and texture, various scales and complex spatial structures of buildings. For example, building roofs have a variety of colors and shapes and are covered by water towers, signal stations, vegetation and other objects. Inter-class differences refer to the spectral and spatial structure differences between buildings and non-buildings, such as the relatively regular shape of buildings compared with natural features. When the proportion of buildings in an image is low, the spectral and spatial structure difference inside buildings is small, which means that the classification accuracy is more affected by inter-class differences (see Figure 4a). When the proportion of buildings in an image is high, the classification accuracy is more affected by intra-class differences. Overall, when exploring potential relationships between the Boltzmann entropy of images and F1, the proportions of buildings in images should be considered.

Based on this, a strategy is proposed here by us:

(1) Analyzing the effects of building proportions on F1;
(2) Exploring relationships between Boltzmann entropy and F1.

## 3. Exploring Relationships between Boltzmann Entropy and F1

### 3.1. Analyzing Effects of Buildings Proportions on F1

**Figure 5:**
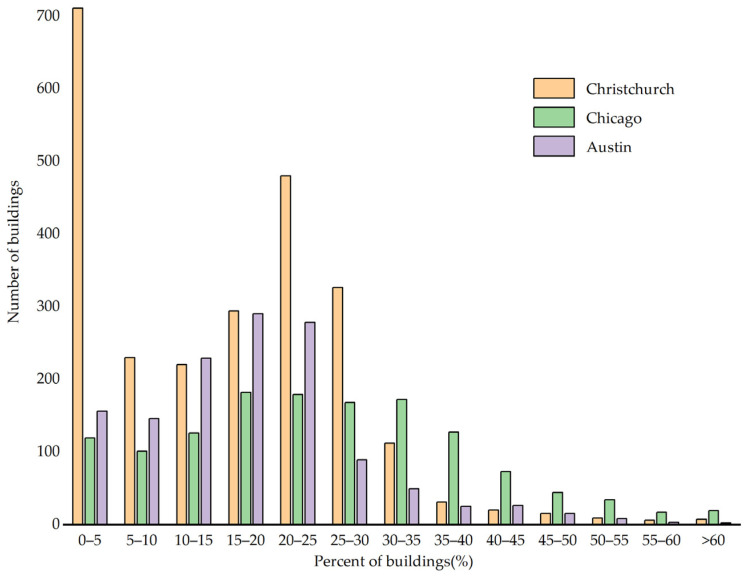
Frequencies of images with different building proportions.

Based on images of three cities (i.e., Christchurch, Chicago and Austin) from the WHU and Inria datasets, the image frequencies by building proportions are shown in Figure 5. It is found that most images have small building proportions (<=35%), while those with large building proportions are few. This is because buildings are often found around artificial and natural elements. In downtown areas, buildings are often separated by roads and other facilities (e.g., greenbelts), while in the suburbs, farmlands and forests dominate.

**Figure 6:**
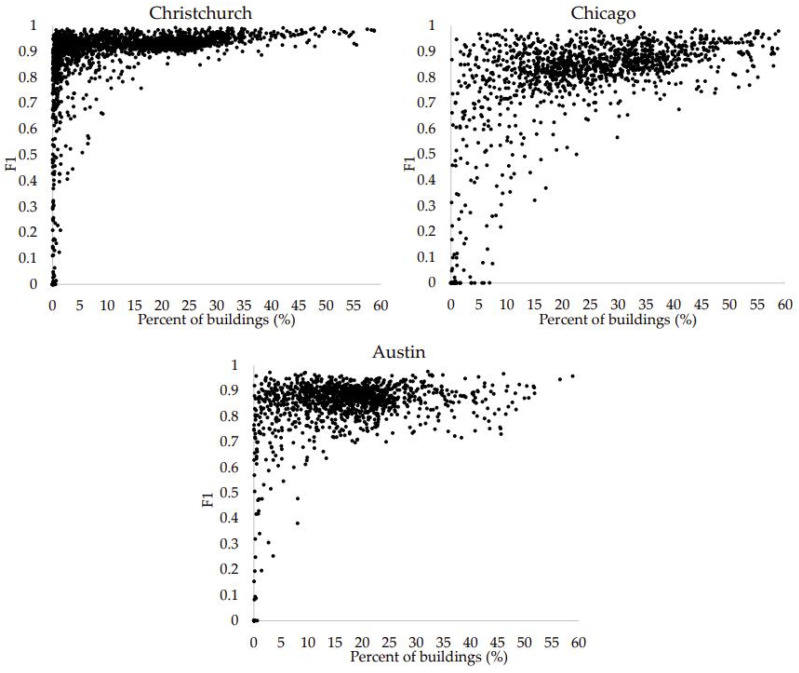
Relationships between building proportions and F1.

In Figure 6, the relationships between building proportions and F1 are given. It is found that when the proportions are small, the fluctuation in F1 is between 0 and 1. With the gradual increase in building proportion, the gap of fluctuation becomes smaller quickly. In particular, when the proportion is larger than 20%, the F1 of images is larger than 0.8. In fact, some factors (e.g., image classifier performance and inaccurate image labeling) will lead to the misclassification of buildings. Regardless of whether the proportion of buildings in an image is large or small, pixels incorrectly classified by these factors exist. In those images with small building proportions, the total numbers of building pixels are small, indicating these incorrectly classified pixels have more impact on F1.

Therefore, to better explore potential relationships between Boltzmann entropy and F1, obvious fluctuation in F1 in images with a specific building proportion should be avoided. To solve this problem, images with very small building proportions are abandoned in the subsequent experiments for exploring potential relationships. The analysis of how images with small building proportions affect the relationships between BE and F1 is presented in Section 4.2.

### 3.2. Relationships between Boltzmann Entropy and F1: Experimental Results

**Figure 7:**
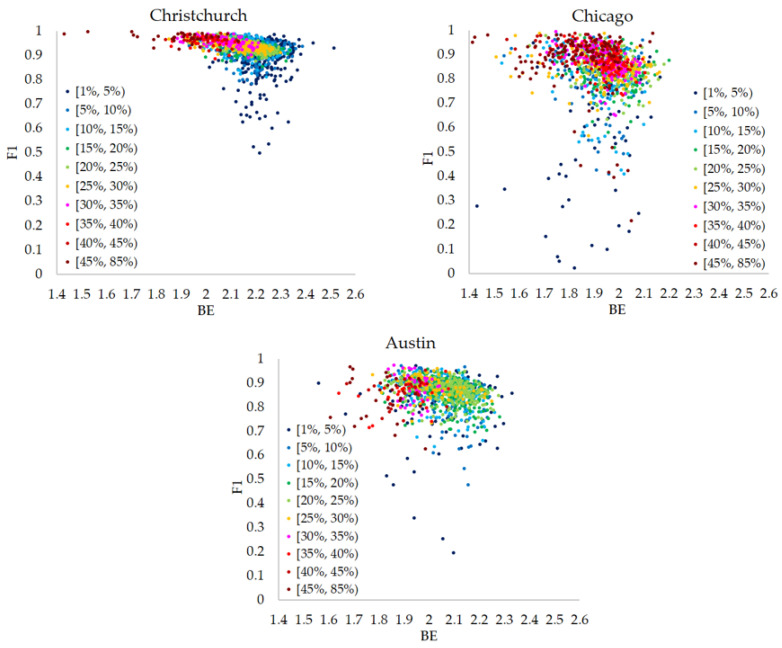
Scattered data of images with building proportions larger than 1%.

All the scattered data between Boltzmann entropy and F1 based on images of three cities are given in Figure 7. Just as we mentioned in Section 3.1, building proportions have effects on F1. To better analyze the relationships, the trends between Boltzmann entropy and F1 under different building proportions are highlighted in different colors in Figure 7.

Correlation analysis is employed to analyze the overall trends, and it is found that the values of Spearman’s correlation coefficients (SCCs) between BE and F1 in three cities are −0.448, −0.259 and −0.252, respectively. As the pairs (BE, F1) are not independent observations, statistical tests are not suitable for this study. To further confirm the possible relationships, various confidence intervals of SCCs are calculated using bootstrapping (i.e., a nonparametric statistical method). It should be noted that when calculating confidence intervals, the number of iterations is set as 1000 (i.e., 1000 sets of paired resamples are generated from the original paired observations), and the size of paired resamples is the same as that of the original paired observations. In addition, the extracted elements are replaced after each sampling. The results of confidence intervals are presented in Table 1. The width of a confidence interval can reflect the degree of uncertainty in our estimate of the Spearman’s correlation coefficient. We can see that the calculated confidence intervals are not very wide (the maximum width is around 0.1), and all the lower and upper bounds of confidence intervals are negative. Such results imply that BE does have a negative relationship with F1.

## 4. Discussion

### 4.1. Possible Upper and Lower Limits of Modeled Relations

**Figure 8:**
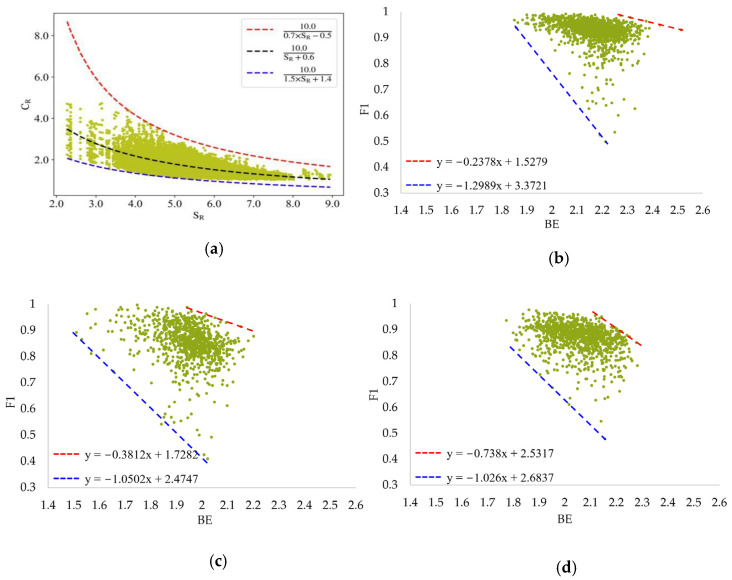
Comparison of modeled relationships based on Boltzmann entropy. (**a**) Relationships between Boltzmann entropy according to resampling ($S_{R}$) and compression ratio ($C_{R}$) [30]; (**b**) relationships between BE and F1 (Christchurch); (**c**) relationships between BE and F1 (Chicago); (**d**) relationships between BE and F1 (Austin).

Recently, some researchers have found that the relationships between Boltzmann entropy and the compression ratio of images can be described by upper and lower limits [30]. The modeled relationships between Boltzmann entropy and the compression ratio are shown in Figure 8a. Naturally, we wonder whether upper and lower limits are appropriate in this study for describing the potential relationships of remote sensing images with entropy and F1. In Figure 8b–d, the modeled relationships with upper and lower limits in three cities are presented. These upper and lower limits are fitted using scatter points near the edges.

We can see that although these limits are different in Christchurch, Chicago and Austin, the modeled lines can basically depict the boundary. Such a result indicates that upper and lower limits are applicable. In fact, because of the lack of data, the scattered points in these figures do not match well with the limits. This means that if we have adequate data, the modeled relationships in these cities may have the same upper and lower limits. In the future, by using more open-source high-resolution remote sensing building datasets, the potential upper and lower limits can be better confirmed.

### 4.2. Effects of Neglecting Images with Small Building Proportions

**Figure 9:**
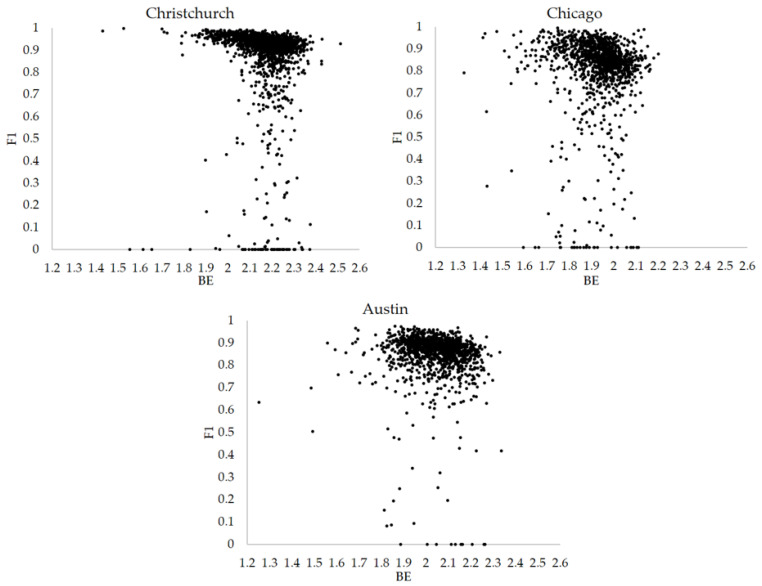
Scatter data with outliers (i.e., images with building proportions smaller than 1%).

The scattered data of images with very small building proportions (i.e., smaller than 1%) are shown in Figure 9, and the SCCs and various confidence intervals are calculated (see Table 2). In terms of SCCs presenting the overall trends, it is found that SCCs change from −0.395, −0.217 and −0.221 to −0.448, −0.259 and −0.252 in three cities if images with building proportions smaller than 1% are removed. In fact, the change is not very large, and in both two situations, negative relationships are all found under various confidence intervals.

## 5. Conclusions

Buildings are important artificial features in remote sensing images and play important roles in many applications. Classifying buildings quickly and accurately is the first step for detailed applications. It has been noticed by many researchers that building classification accuracy is greatly affected by image quality. However, the potential relationships between image quality and building classification accuracy have not been modeled.

In this study, remote sensing images from two open-source datasets (i.e., WHU and Inria) in three cities (i.e., Christchurch, Chicago and Austin) are employed, and potential relationships between Boltzmann entropy (an image quality index considering both compositional and configurational information) and F1 (a comprehensive metric considering both precision and recall rate) are explored. Experimental results show that F1 fluctuates greatly in images with small building proportions (especially in images with building proportions smaller than 1%). In addition, negative relationships between BE and F1 are found using correlation analysis (the SCCs are −0.448, −0.259 and −0.252, respectively, in the three cities). Such negative relationships are confirmed by various confidence intervals calculated using bootstrapping in both situations (i.e., with and without images that have building proportions smaller than 1%). The maximum width of all confidence intervals is around 0.1, and all lower and upper bounds of confidence intervals are negative. From the above results, we may conclude that the Boltzmann entropy of remote sensing images does have a negative relationship with F1 (i.e., when BE becomes larger, the F1 tends to become smaller). These discoveries are helpful to improve our understanding of how image quality affects building classification accuracy.

There are some limitations of this study. The first is that the employed images are only very-high-resolution ones, and the image object type is restricted to buildings. Thus, we cannot conclude whether potential relationships exist for images with different resolutions and objects (e.g., roads). Secondly, the effects of the size of clipped images on the classification are not considered in this study. In fact, this may have impacts on the exploration of subsequent relationships.

## Figures and Tables

**Table 1:** Confidence intervals of SCCs calculated using bootstrapping.

Confidence Interval			
	Christchurch (Image Number: 2114 SCC = −0.448)	Chicago (Image Number: 1269 SCC = −0.259)	Austin (Image Number: 1245 SCC = −0.252)
95%	(−0.485, −0.407)	(−0.313, −0.207)	(−0.307, −0.196)
90%	(−0.479, −0.413)	(−0.304, −0.218)	(−0.297, −0.206)
85%	(−0.475, −0.417)	(−0.298, −0.222)	(−0.293, −0.213)
80%	(−0.473, −0.422)	(−0.295, −0.226)	(−0.288, −0.218)
75%	(−0.470, −0.425)	(−0.292, −0.229)	(−0.284, −0.222)
70%	(−0.468, −0.427)	(−0.288, −0.232)	(−0.282, −0.225)
65%	(−0.466, −0.429)	(−0.285, −0.235)	(−0.279, −0.229)
60%	(−0.464, −0.431)	(−0.282, −0.237)	(−0.276, −0.231)
55%	(−0.463, −0.433)	(−0.280, −0.239)	(−0.273, −0.233)
50%	(−0.461, −0.435)	(−0.278, −0.241)	(−0.271, −0.235)
45%	(−0.460, −0.436)	(−0.275, −0.243)	(−0.269, −0.237)
40%	(−0.458, −0.438)	(−0.273, −0.245)	(−0.267, −0.238)
35%	(−0.456, −0.440)	(−0.271, −0.247)	(−0.266, −0.240)
30%	(−0.455, −0.441)	(−0.269, −0.248)	(−0.264, −0.242)
25%	(−0.454, −0.442)	(−0.268, −0.250)	(−0.262, −0.244)
20%	(−0.452, −0.443)	(−0.266, −0.252)	(−0.260, −0.245)
15%	(−0.452, −0.444)	(−0.264, −0.254)	(−0.258, −0.247)
10%	(−0.450, −0.445)	(−0.262, −0.255)	(−0.256, −0.249)
5%	(−0.449, −0.446)	(−0.261, −0.257)	(−0.254, −0.251)

**Table 2:** Results for SCCs and various confidence intervals of images with building proportions smaller than 1%.

Confidence Interval			
	Christchurch (Image Number: 2415, Including 301 Images with Building Proportions Smaller than 1% SCC = −0.395)	Chicago (Image Number: 1311, Including 42 Images with Building Proportions Smaller than 1% SCC = −0.217)	Austin (Image Number: 1301, Including 56 Images with Building Proportions Smaller than 1% SCC = −0.221)
95%	(−0.434, −0.356)	(−0.269, −0.164)	(−0.276, −0.168)
90%	(−0.426, −0.364)	(−0.260, −0.170)	(−0.266, −0.176)
85%	(−0.423, −0.366)	(−0.256, −0.176)	(−0.260, −0.182)
80%	(−0.421, −0.369)	(−0.252, −0.180)	(−0.255, −0.187)
75%	(−0.418, −0.372)	(−0.248, −0.184)	(−0.253, −0.190)
70%	(−0.416, −0.374)	(−0.246, −0.187)	(−0.250, −0.194)
65%	(−0.414, −0.376)	(−0.243, −0.191)	(−0.247, −0.197)
60%	(−0.411, −0.378)	(−0.241, −0.192)	(−0.245, −0.199)
55%	(−0.410, −0.380)	(−0.238, −0.195)	(−0.243, −0.202)
50%	(−0.408, −0.382)	(−0.235, −0.198)	(−0.240, −0.203)
45%	(−0.407, −0.384)	(−0.233, −0.201)	(−0.238, −0.205)
40%	(−0.405, −0.386)	(−0.231, −0.202)	(−0.235, −0.207)
35%	(−0.404, −0.387)	(−0.230, −0.205)	(−0.234, −0.209)
30%	(−0.403, −0.389)	(−0.229, −0.207)	(−0.232, −0.211)
25%	(−0.402, −0.390)	(−0.227, −0.209)	(−0.231, −0.213)
20%	(−0.400, −0.391)	(−0.225, −0.210)	(−0.228, −0.215)
15%	(−0.399, −0.393)	(−0.223, −0.212)	(−0.226, −0.216)
10%	(−0.398, −0.393)	(−0.222, −0.215)	(−0.225, −0.218)
5%	(−0.397, −0.395)	(−0.220, −0.217)	(−0.223, −0.220)

## Data Availability

The datasets employed in this study are open-source.
